# Design, Simulation and High Precision Tracking Control of a Piezoelectric Optical Stabilization Platform

**DOI:** 10.3390/mi17010087

**Published:** 2026-01-08

**Authors:** Yonggang Yan, Can Cui, Jianjun Cui, Fuming Zhang, Kai Chen, Junjie Huang, Hang Xie, Dengpan Zhang

**Affiliations:** 1 School of Mechanical and Power Engineering, Henan Polytechnic University, Jiaozuo 454003, China; 212305010037@home.hpu.edu.cn (C.C.); anny@hpu.edu.cn (J.H.); 13838747507@163.com (H.X.); zhangdengpan@hpu.edu.cn (D.Z.); 2 National Institute of Metrology, Beijing 100029, China; ycuijj@163.com; 3 State Key Laboratory of Precision Measuring Technology and Instruments, Tianjin University, Tianjin 300072, China; zhangfumin@tju.edu.cn

**Keywords:** optical image stabilization, piezoelectric actuators, hysteresis compensation, precision control

## Abstract

Optical image stabilization (OIS) is crucial for improving airborne opto-electronic imaging performance under dynamic conditions. This study presents a two-dimensional piezoelectric-driven OIS platform capable of compensating linear image shift errors. A motion platform integrating a bridge amplification mechanism and right-angle guiding beams was developed, and its theoretical model was validated through finite element analysis (FEA). To enhance the platform’s repeatability, the hysteresis of the piezoelectric actuator was described using the Bouc-Wen model, and was optimized using a Hybrid Genetic Algorithm and Particle Swarm Optimization (HGAPSO). Experimental results demonstrated that the platform achieves a workspace of 53.92 μm × 53.76 μm, a motion resolution of 30 nm, a maximum coupling error of 2.28%, and a first-order resonant frequency of 356.69 Hz. A composite controller incorporating HGAPSO attained submicron tracking accuracy, with errors of 0.43 μm and 0.47 μm along the X and Y axes, respectively. Strong agreement among theoretical analysis, FEA, and experimental results confirms the platform’s precision and effectiveness meeting the requirements of the OIS. This work provides valuable guidance for the development of high-frequency OIS systems in highly dynamic operational environments.

## 1. Introduction

High-precision optical imaging is a cornerstone for scientific research and industrial applications, enabling breakthroughs in areas such as space exploration [1], biomedical diagnostics [2,3], remote sensing [4,5], and nanoscale metrology [6]. However, optical imaging systems, working in dynamic environments, have been facing great challenges: platform vibrations, attitude fluctuations, and mechanical perturbations collectively degrading imaging resolution. This issue becomes particularly critical in submicron- or nanometer-scale imaging missions, where even micro jitters induce pronounced image blurring or distortion, severely compromising system fidelity. Consequently, advancing high-resolution optical stabilization techniques for airborne platforms has emerged as an urgent technological priority.

Conventional stabilization approaches, such as mechanical platforms, electronic algorithms, and optical compensation, exhibit some critical limitations [7]. The mechanical systems with gyroscope-based servo control systems are too bulky, have slow response times and depend on high-performance inertial sensors, which makes them unsuitable for high-frequency perturbation compensation. Electronic stabilization algorithms mitigate motion artifacts through post-processing, and they can introduce computational latency and geometric distortions, restricting utility in real-time dynamic scenarios [8]. Optical stabilization methods, which directly adjust optical components via actuators, offer superior performance in low-light and long-focal-length applications by preserving native image quality [9]. Nevertheless, existing optical systems face implementation barriers due to structural complexity, prohibitive costs, and inadequate robustness in high-disturbance environments. These constraints underscore the need for innovative optical stabilization platforms tailored to airborne optoelectronic systems.

The vibration characteristics of airborne platforms are usually characterized by low-frequency high-amplitude, high-frequency low-amplitude and fast dynamic perturbations, which poses a serious challenge to the image stabilization performance of the optoelectronic system. The integrated system of piezoelectric actuator (PEA) and flexible mechanism is widely used due to its advantages of high stiffness, high resolution and high dynamic response [10]. NASA used PEA to achieve submicron OIS in space telescopes, which significantly improved the perturbation resistance of the imaging system [11]. Japanese scholars proposed an adaptive OIS method based on PEA, which effectively improves the stability of the imaging system by detecting and compensating external perturbations in real time [12]. Ling et al. demonstrated millimeter-scale motion (1.21 mm × 1.21 mm) using rhombic lever amplifiers, though limited by sub-50 Hz bandwidth [13]. Xiao et al. proposed a hybrid XYZΘ positioning mechanism capable of a translational motion of 204.2 μm × 212.8 μm and a rotation of 8.7 mrad with a coupling error of less than 3%, and its first-order intrinsic frequency was 87.8 Hz [14]. Wu-Le Zhu et al. used two Scott -Russell and half-bridge mechanism combination to amplify the displacement of the PEA, which was decoupled by a leaf-shaped double parallelogram structure to achieve good decoupling characteristics, and a closed-loop controller was used to achieve a tracking error within 0.5 μm [15].

Recent advances focus on two frontiers. One is compensation of piezoelectric nonlinearities (hysteresis, creep) through advanced constitutive modeling, and the other is development of multi-degree-of-freedom (MDOF) stabilization platforms. The Bouc-Wen (BW) model is a popular method used to model the hysteresis behavior of piezoelectric ceramics (PZT). This model requires additional nonlinear differential equations to accurately describe the hysteresis ring [16,17]. The BW model has several benefits, including requiring fewer parameters, being easy to implement, and having an inverse model that is easy to solve. Zhang et al. developed an asymmetric BW model for piezoelectric-actuated ball screws in swash plate pumps, achieving dynamic stiffness compensation, which reduced swash plate oscillations by 94% during displacement switching [18]. Qian et al. proposed a modified BW model, which can adapt to various excitation conditions and achieve an average relative error within 4% under different current excitations [19]. Yin et al. predicted the shear motion trajectory of a piezoelectric stack by using the electric field and frequency identified by BW, and the prediction error rate of hysteresis loop amplitude was less than 16.5%, with a minimum value of 0.1% [20]. Chang et al. developed a MDOF platform with adaptive control algorithms reducing perturbation-induced errors, but there is still a need for integration and miniaturization [21]. Despite these achievements, three fundamental challenges persist. For example, it is often difficult for the existing platforms to take into account the high intrinsic frequency while achieving large travel, which limits their application in high-frequency perturbation environments; the influence of the nonlinear characteristics of PZT (e.g., hysteresis and creep) on the control accuracy still needs to be further researched and compensated; and how to achieve low-coupling and high-precision control among the degrees of freedom in the multi-degree-of-freedom stabilized-image platforms still remains a technological difficulty, etc. [22].

To address existing trade-offs, this study presents a piezoelectric-driven optical stabilization platform founded on three integrated innovations: (1) a symmetrical mechanical design merging a bridge amplifier with orthogonal L-shaped guides to simultaneously deliver a large workspace (>50 µm) and high resonant frequency (>350 Hz); (2) a hysteresis compensation strategy using an HGAPSO-identified Bouc-Wen model to enable submicron positioning; and (3) a composite HGAPSO-feedforward/PID-feedback control scheme achieving submicron tracking accuracy (0.43–0.47 µm) with effective decoupling. This synergistic approach provides a comprehensive solution for high-precision stabilization in dynamic environments. The main research work includes: Section 2 introduces the structural design of the optically stabilized platform and the theoretical analysis of the platform, including static and dynamic modeling; Section 3 adopts the BW model for the piezoelectric hysteresis problem and uses the HGAPSO to identify the parameters of the model; and in Section 4, the measurement and control system of the platform is established, and the open-loop travel test, intrinsic frequency test, resolution test, controller design and trajectory tracking experiments are carried out.

## 2. Structural Design and Simulation

### 2.1. Description of Mechanical Architecture

**Figure 1:**
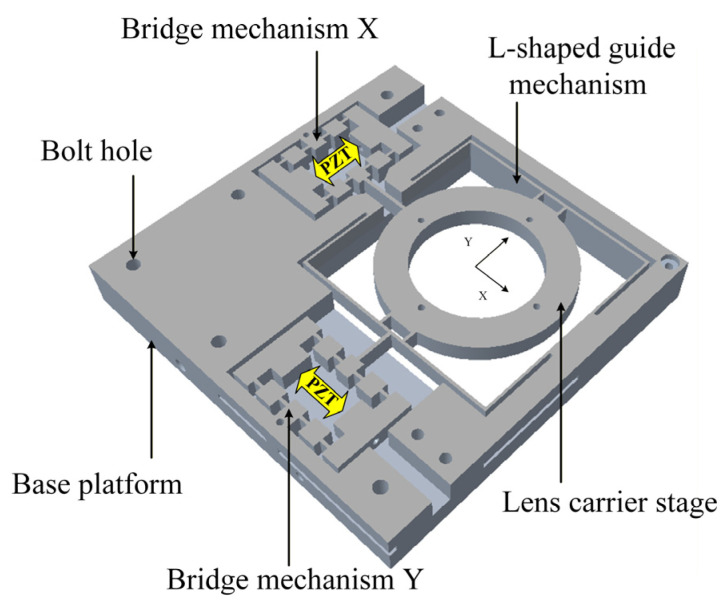
Schematic of the XY optical stabilization stage.

As illustrated in Figure 1, the optical stabilization platform integrates a bridge-type displacement amplification mechanism with orthogonally arranged L-shaped guidance beams, forming a compact dual-layer structure.

The upper functional layer comprises a lens carrier stage and a PZT and is rigidly coupled to the lower base via bolted interfaces. The system’s operation is achieved through the coordinated action of three key subsystems: piezoelectric actuators generate bidirectional displacements with controlled voltages; the bridge mechanism amplifies these motions via elastic hinge deformation; and leaf-spring-based right-angle guides simultaneously stabilize the lens carrier stage while suppressing parasitic displacements in non-target axes. By modulating input voltages to the XY-oriented piezoelectric elements dynamically, the system precisely transfers amplified displacements to the centrally mounted compensation lens, thereby counteracting image shifts through real-time optomechanical adjustments. This electromechanical synergy enables submicron-scale stabilization while maintaining structural rigidity and motion decoupling. The symmetrical integrated design is adopted for the two directions of movement of the mechanism, avoiding unnecessary processing errors and coupling errors. The mechanism has some great advantages of the compactness and symmetrical output.

The specific design index of the optical stabilization platform is shown in Table 1.

Considering the requirements for lightness, stability, and corrosion resistance, the piezoelectric optical stabilization platform is made of 7075 aluminum alloy, with specific material property parameters listed in Table 2. The piezoelectric ceramic actuator used is Pst150/5 × 5/20H (Harbin Core Tomorrow Science & Technology Co., Ltd., Harbin, China), with a nominal maximum stroke of 20 μm, a stiffness of 60 N/μm, and detailed parameters provided in Table 3.

### 2.2. Theoretical Analysis of the Platform

#### 2.2.1. Static Modeling

**Figure 2:**
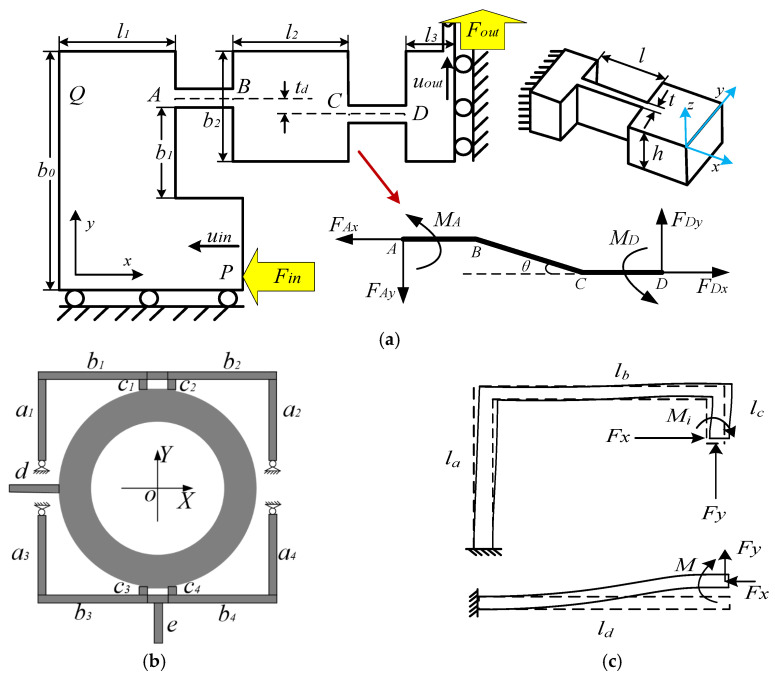
Mechanical structural design: (**a**) 1/4 bridge and flexible hinge; (**b**) guide mechanism structure diagram; (**c**) the deformation of the guiding beam and the driving beam.

To establish a comprehensive performance model of the optical stabilization platform, a hydrostatic analytical framework is developed based on Euler-Bernoulli beam theory and Castigliano’s second theorem [23,24]. The bridge-type displacement amplification mechanism, comprising eight serially parallel leaf flexures, is analyzed through a quarter-symmetry submodule (Figure 2a) to derive its kinematic and static characteristics.

The leaf-shaped flexible hinges AB and CD can be viewed as a simple beam unit, assuming no motion in the Z direction, which produces a generalized displacement of $X_{i}={\left[\begin{array}{ccc} d_{x} & d_{y} & \theta_{z} \end{array}\right]}^{T}$ with a generalized external force $\bar{F}={\left[\begin{array}{ccc} F_{x} & F_{y} & M_{z} \end{array}\right]}^{T}$ onto the free end of the beam. The relationship between the displacement and the external force can be expressed by the following Equation (1):

(1) $$X_{i}=C\bar{F}$$

where *C* denotes the flexibility matrix of the unit’s flexible hinge in the local coordinate system. And the matrix of its flexibility coefficients is given in literature [25], expressed by Equation (2):

(2) $$C=\left[\begin{array}{ccc} \frac{\partial d_{x}}{\partial F_{x}} & 0 & 0\\ 0 & \frac{\partial d_{y}}{\partial F_{y}} & \frac{\partial d_{y}}{\partial M_{z}}\\ 0 & \frac{\partial \theta_{z}}{\partial F_{y}} & \frac{\partial \theta_{z}}{\partial M_{z}} \end{array}\right]=\left[\begin{array}{ccc} \frac{l}{Eht} & 0 & 0\\ 0 & \frac{4l^{3}}{Eht^{3}} & \frac{6l^{2}}{Eht^{3}}\\ 0 & \frac{6l^{2}}{Eht^{3}} & \frac{12l}{Eht^{3}} \end{array}\right]$$

where *E* is the modulus of elasticity of the material and *t*, *h* and *l* are the geometrical parameters of the hinge. This matrix describes the local deformation of an individual flexure hinge. Due to the symmetrical layout of the mechanism, the overall static and dynamic coupling characteristics between the X and Y translational degrees of freedom are designed to be identical and minimal.

Assuming that PZT applies a horizontal input force *F_in_* at the input *P* of the whole bridge mechanism, the hinges AB, CD and the parts other than the connecting block BC are regarded as rigid bodies. When the mechanism is in equilibrium, BC is overturned, the output force of the mechanism is *F_out_*, and the angular displacements of points *A* and *D* are zero. Based on the schematic configuration, force and moment equilibrium equations are established for beams AB, BC, and CD. Applying Castigliano’s second theorem, the strain energy within the ABCD segment is related to its deformation displacement through the following energy-displacement relationship:

(3) $$\left\{\begin{array}{c} u_{in}=\sum_{i=AB,BC,CD}\left[\int_{l_{i}}\frac{F_{i}(x_{i})}{EA_{i}}\frac{\partial F_{i}(x_{i})}{\partial F_{x}}dx+\int_{l_{i}}\frac{M_{i}(x)}{EI_{i}}\frac{\partial M_{i}(x_{i})}{\partial F_{x}}dx\right]\\ u_{out}=\sum_{i=AB,BC,CD}\left[\int_{l_{i}}\frac{F_{i}(x_{i})}{EA_{i}}\frac{\partial F_{i}(x_{i})}{\partial F_{y}}dx+\int_{l_{i}}\frac{M_{i}(x)}{EI_{i}}\frac{\partial M_{i}(x_{i})}{\partial F_{y}}dx\right] \end{array}\right.$$

where *F_i_* and *M_i_* are the axial force and moment equations for the corresponding beam *i*, respectively, and *A_i_* and *I_i_* denote the cross-sectional area and inertia distance of the corresponding beam *i*.

Substituting the force and moment equations of the beams into Equation (3), the following relationship between the input force and the output displacement of the bridge mechanism is obtained as follows:

(4) $$\left(\begin{array}{c} u_{in}\\ u_{out} \end{array}\right)=C_{r}\left(\begin{array}{c} F_{in}\\ F_{out} \end{array}\right)=\left[\begin{array}{cc} c_{11} & c_{12}\\ c_{21} & c_{22} \end{array}\right]\left(\begin{array}{c} F_{in}\\ F_{out} \end{array}\right)$$

where *Cr* is the flexibility matrix of the bridge mechanism, which is achieved by transforming the flexibility matrix of each hinge to the global coordinate system through standard rotation and translation transformations, and then summing them. Here,

$$c_{11}=\frac{2lt^{2}+6lt_{d}}{Eht^{3}}+\frac{l_{2}b_{2}^{2}+l_{2}t_{d}^{2}}{Ehb_{2}^{3}};\;c_{12}=c_{21}=\frac{6l(l+l_{2})t_{d}}{Eht^{3}}+\frac{l_{2}t_{d}}{Ehb_{2}^{3}};\;c_{22}=\frac{(8l^{3}+12l^{2}l_{2}+6ll_{2}^{2})t_{d}}{Eht^{3}}+\frac{l_{2}^{3}}{Ehb_{2}^{3}}.$$

According to the definition of input-output stiffness as well as magnification, the theoretical values of input-output stiffness and magnification of the bridge mechanism can be calculated by making the input and output forces equal to 0 in Equation (4).

(5) $$\left\{\begin{array}{l} K_{in}=\frac{F_{in}}{2u_{in}}=\frac{1}{2c_{11}}\\ K_{out}=\frac{F_{out}}{u_{out}}=\frac{c_{11}}{c_{11}c_{22}-c_{12}c_{21}}\\ Amp=\frac{u_{out}}{u_{in}}=\frac{c_{21}}{c_{11}} \end{array}\right.$$

The amplified displacements from the XY bridge mechanism are transmitted through an orthogonal guidance system to drive the lens platform. As illustrated in Figure 2b, this system comprises four L-shaped guiding beams (*a_i_*,*b_i_*,*c_i_*), with beams d and e serving as bidirectional push rods. Under small-deformation conditions, the displacements at beam ends *c*_1–4_ dominate the platform output, allowing negligible deformation approximation for short beams *b*_1_*b*_2_ and *b*_3_*b*_4_. The force distribution diagram in Figure 2c depicts the loading conditions on guide beam *a_i_*,*b_i_*,*c_i_* and push beam *d*, with symmetric loading assumed across all four quadrants.

When subjected to orthogonal platform forces *F_x_* and *F_y_*, each guiding beam segment (*a_i_*,*b_i_*,*c_i_*) develops characteristic axial forces and bending moments. Applying Hooke’s law while neglecting torsional effects, these force-moment relationships are substituted into Equation (3) to derive the flexibility matrix for a single L-shaped guide beam in principal axes:

(6) $$\left\{\begin{array}{l} K_{gx}^{-1}=C_{gx}=\frac{{\left(l_{a}+l_{c}\right)}^{3}-l_{a}^{3}}{3EI_{a}}+\frac{l_{b}}{EA_{b}}+\frac{l_{b}l_{c}^{2}}{EI_{b}}+\frac{l_{c}^{3}}{3EI_{c}}\\ K_{gy}^{-1}=C_{gy}=\frac{l_{a}}{EA_{a}}+\frac{l_{a}l_{b}^{2}}{EI_{a}}+\frac{l_{b}^{3}}{3EI_{b}}+\frac{l_{c}}{EA_{c}} \end{array}\right.$$

Assuming that the maximum output stroke of the PZT with a stiffness of *K_pzt_* is *u_max_*, the input displacement of the bridge mechanism is

(7) $$u_{pzt}=\frac{K_{pzt}}{K_{pzt}+K_{in}}u_{\max}$$

Consider the guide mechanism and the motion platform as load outputs from the bridge mechanism. According to the series-parallel relationship of the mechanism and combining with Equations (5)–(7), the maximum travel of the image stabilizing platform in the X and Y directions is found to be

(8) $$\left\{\begin{array}{l} X_{p}=Amp\cdot u_{pzt}\cdot (\frac{K_{out}}{K_{out}+K_{px}})\\ Y_{p}=Amp\cdot u_{pzt}\cdot (\frac{K_{out}}{K_{out}+K_{py}}) \end{array}\right.$$

where *K_px_*, *K_py_* denote the combined stiffness of the guiding mechanism and the push beam in the X and Y directions.

Based on the design specifications in Table 1, the stiffness calculated from the theoretical model, and constraints such as stress and dimensions, the dimensional parameters of the platform were optimized. The final dimensional results are presented in Table 4, where the parameters correspond to the geometric structure shown in Figure 2. With these dimensions, the theoretical model Equation (8) yields a maximum travel of 54.09 μm × 54.48 μm, satisfying the designed stroke requirement.

#### 2.2.2. Kinetic Model Analysis

The design of optical stabilization stages necessitates careful consideration of intrinsic frequency characteristics, particularly for piezoelectric micro positioning systems where dynamic performance is governed by Lagrangian mechanics. Given the symmetric mass distribution and kinematic similarity in orthogonal axes, the system’s fundamental frequency is analyzed through a reduced-order model considering only the X-direction dynamics, with the Y-direction assumed stationary and gravitational effects neglected. Under this formulation, the generalized coordinate d represents the input displacement, with corresponding kinetic (*T*) and potential (*V*) energy terms incorporated into the Lagrangian framework [26,27]:

(9) $$\frac{d}{dt}\frac{\partial T}{\partial \bar{d}}-\frac{\partial T}{\partial d}+\frac{\partial V}{\partial d}=F$$

where *F* is the input force corresponding to the displacement *d* in the X direction.

The first order intrinsic frequency of the device can be calculated as

(10) $$f=\frac{1}{2\pi }\sqrt{\frac{K}{M}}$$

### 2.3. Finite Element Simulation and Its Analysis

**Figure 3:**
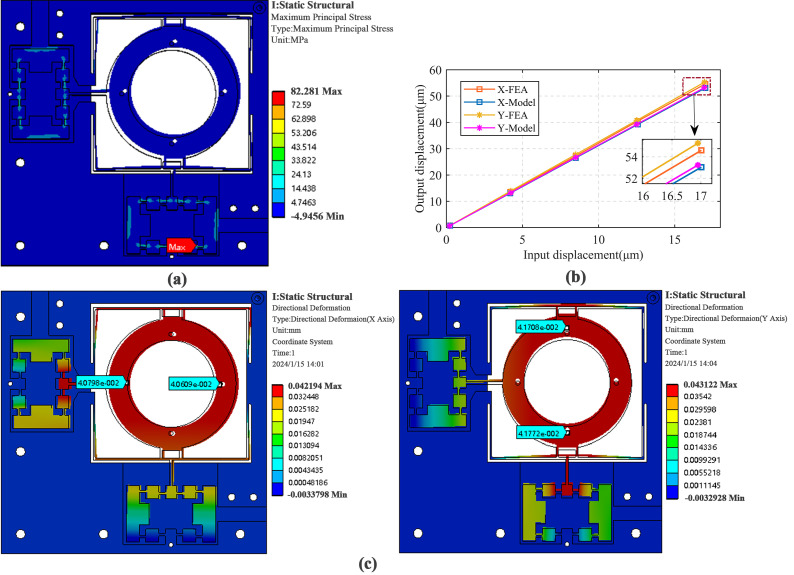

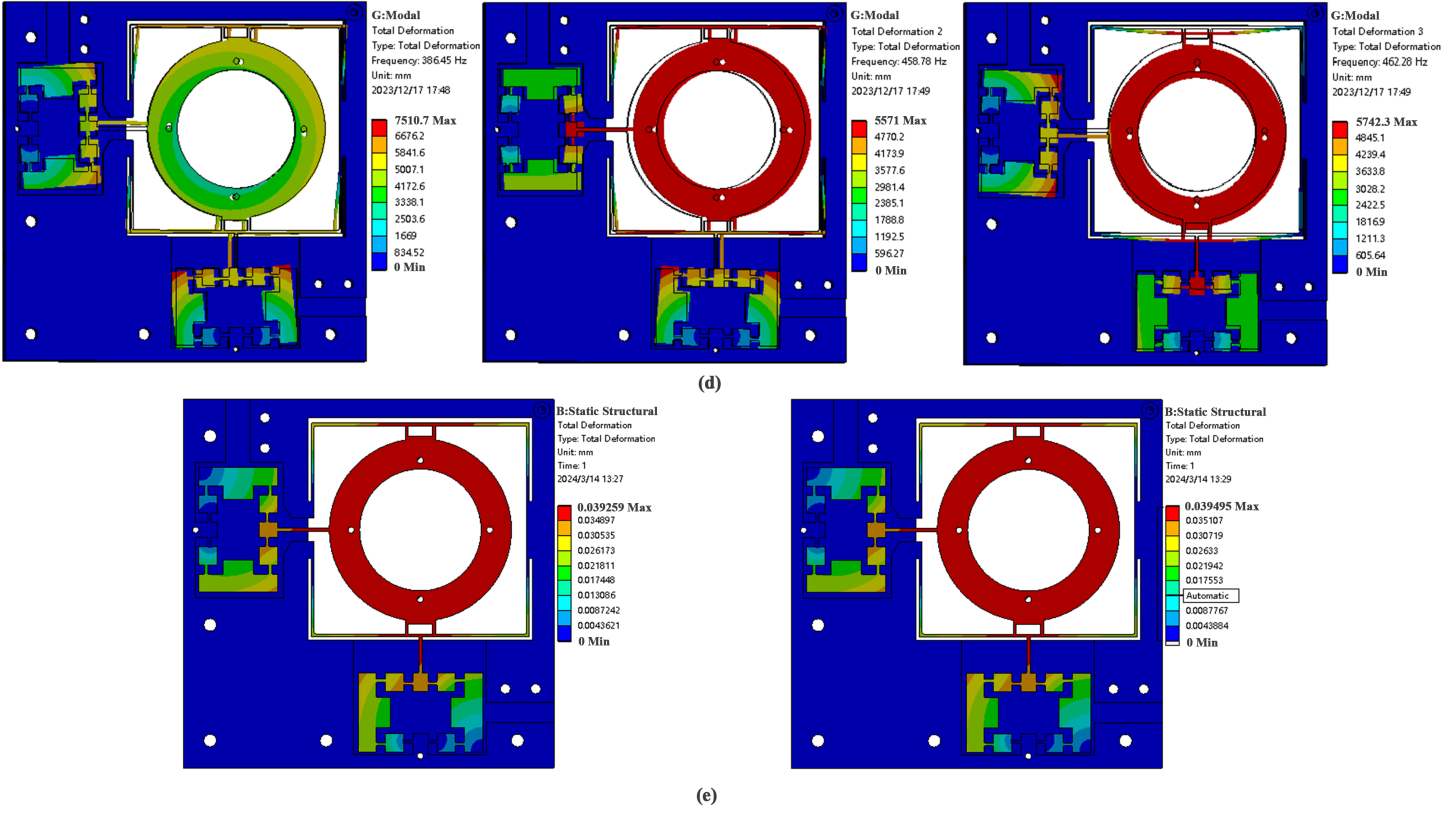
Finite element simulation results. (**a**) Structural strain simulation; (**b**) theoretical models and FEA; (**c**) displacement output simulation; (**d**) first 3 orders of FEA mode; (**e**) load capacity simulation.

To evaluate the structural integrity and operational stability of the optimized optical stabilization platform, the strength and strain of the platform is simulated in ANSYS Workbench 2020 R2 software. As depicted in Figure 3a, bidirectional input forces of 150 N are applied to simulate operational stresses, inducing positive X and Y displacements at the lens platform. The stress distribution analysis reveals maximum von Mises stresses of 82.28 MPa localized at the bridge mechanism’s flexure hinges—well below the material yield strength—confirming elastic deformation dominance throughout the operational range and ensuring structural safety.

The FEA results are systematically compared against theoretical predictions across multiple loading conditions (3 N, 50 N, 150 N, 200 N). Figure 3b illustrates the input-output relationships for both models in orthogonal axes, demonstrating excellent linearity within the PEA’s operational range. The maximum discrepancies between FEA and theoretical predictions are limited to 2.86% (X-axis) and 3.67% (Y-axis), validating the theoretical model’s accuracy. Minor deviations primarily stem from simplifications in theoretical derivations, particularly the neglect of secondary deformations in flexure elements, resulting in slightly conservative displacement predictions.

Finite element simulation analyses of the travel of the optical stabilization stage are carried out. Unidirectional loading simulations (150 N) are performed to characterize displacement amplification (Figure 3c). The X-axis configuration yields 40.70 μm average displacement with 3.22× amplification, while the Y-axis exhibited 41.74 μm displacement at 3.27× amplification. Due to the non-completely symmetric parallel structure, the device generates a smaller parasitic motion between two directions, which leads to the maximum displacement. Parasitic motion, resulting in the maximum displacement occurring at the right angle of the guiding mechanism furthest from the bridge mechanism, and the output error at both ends of the diameter direction is less than 0.5% in both cases.

The vibration modes of the OIS platform are simulated and analyzed. Vibration mode simulations (Figure 3d) identified three dominant modes: (1) 386.45 Hz rotational mode about the Z-axis, attributed to residual coupling effects; (2) 458.78 Hz X-axis translational mode; and (3) 462.28 Hz Y-axis translational mode. The close frequency proximity of orthogonal translational modes (Δ*f* = 3.5 Hz) indicates well-balanced dynamic characteristics, enabling consistent stabilization performance across operational axes. These results demonstrate the platform’s capability to compensate image shifts across a broad frequency spectrum.

To investigate the load carrying capability of the platform when carrying a lens, a comparative simulation was performed, as shown in Figure 3e. First, 100 N was applied in both the X and Y directions, resulting in a maximum total displacement of 39.259 µm. Subsequently, a 2 N downward force was applied to the lens carrier stage to simulate the lens load. The total displacement field obtained under the combined loading (right Figure 3e) is 39.495 µm, indicating that the additional load introduces only about 0.236 µm of out-of-plane (Z-direction) displacement, which corresponds to just 0.6% of the unloaded maximum output displacement. Moreover, motion parallel to the optical axis generally has a smaller impact on image degradation compared to motion perpendicular to it. Therefore, the Z-axis deformation under a 2 N load has a negligible influence on the in-plane displacement output, demonstrating that the designed platform possesses good load-bearing capability for practical optical components.

## 3. Control Algorithm Design

### 3.1. Description of Classical BW Model

The operational performance of piezoelectric-driven stabilization stages is fundamentally constrained by inherent nonlinearities, particularly the hysteresis characteristics of PZT actuators [28,29]. To achieve submicron positioning accuracy, a hybrid control architecture integrating BW hysteresis modeling with proportional-integral-derivative (PID) compensation was developed. This approach explicitly addresses hysteresis-induced displacements, enabling precise trajectory tracking and position regulation.

**Figure 4:**
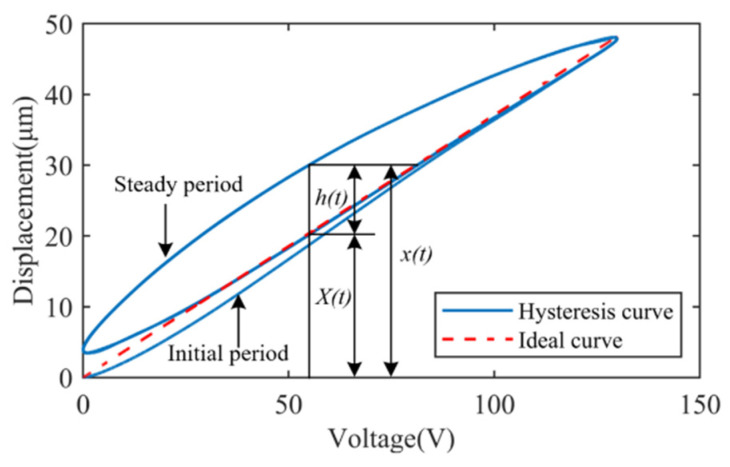
BW Hysteresis curve.

As shown in Figure 4, the BW hysteresis model considers the output displacement *x*(*t*) of the platform as the superposition of the linear component *X*(*t*) and the hysteresis component *h*(*t*), i.e.,

(11) x(t)=X(t)+h(t)

The specific BW model expression is given by

(12) $$\left\{\begin{array}{l} x(t)=ku(t)+h(t)+x_{0}\\ \dot{h}(t)=\alpha \dot{u}(t)-\beta \left|\dot{u}(t)\right|\cdot {\left|h(t)\right|}^{n-1}h(t)-\gamma \dot{u}(t){\left|h(t)\right|}^{n} \end{array}\right.$$

where *x*_0_ is the initial displacement of the platform, *u*(*t*) is the input voltage of the system, is *k* is the ratio parameter of the output displacement to the input voltage, *α* determines the amplitude of the hysteresis curve, *β* and *γ* determine the sharpness of the hysteresis loop, and *n* is the smoothness coefficient of the hysteresis curve, which is usually taken to be 1. To accurately simulate the hysteresis displacements of the PZT, the values of the parameters *k*, *α*, *β*, and *γ* are required to be determined by parameter identification.

### 3.2. Parameter Identification

**Figure 5:**
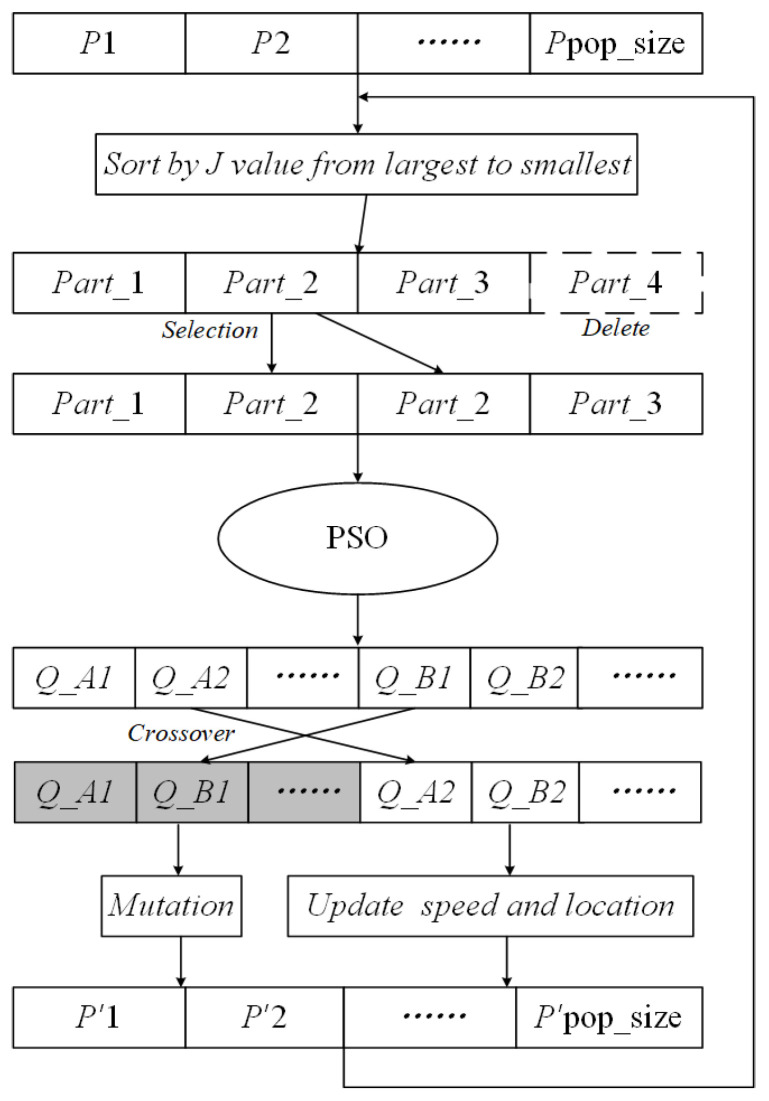
HGAPSO algorithm.

The parameter identification for the BW hysteresis model is implemented through a hybrid metaheuristic algorithm, which synergistically integrates genetic algorithm (GA) and particle swarm optimization (PSO) techniques. As illustrated in Figure 5, the HGAPSO algorithm is initiated with population partitioning into fitness-based quartiles, retaining the top 75% individuals while replicating medium-fitness candidates to maintain genetic diversity. During each iteration, the velocity and position of each solution is refined using PSO-based updates, followed by the stochastic selection of elite candidates from complementary subgroups for real-coded crossover and mutation operations. The resulting offspring are subsequently recombined with PSO-optimized particles, forming a regenerated population for the next evolutionary cycle. This iterative process continues until the convergence criteria are met, combining the global exploration capabilities of GA with the local refinement strengths of PSO to robustly identify BW model parameters, while avoiding premature convergence to local optima.

During the iteration process, the PSO algorithm searches for the velocity and position of the particle with the following equation:

(13) $$\left\{\begin{array}{l} v_{i+1}=wv_{i}+c_{1}r_{1}(P_{i}-x_{i})+c_{2}r_{2}(G_{i}-x_{i})\\ x_{i+1}=x_{i}+v_{i+1} \end{array}\right.$$

where *v_i_* denotes the velocity of the particle before updating, *x_i_* denotes the position of the particle before updating, *w* is the inertia weight, *c*_1_ and *c*_2_ are the learning factors, *r*_1_ and *r*_2_ are random numbers in the range of [0, 1], and *P_i_* and *G_i_* denote the optimal positions of the current individual and population, respectively.

Inertia weights strongly influence search ability. To avoid falling into local optima, let the inertia weights change with the number of iterations, expressed by

(14) $$w=w_{s}-\frac{(w_{s}-w_{e})t}{T}$$

where *w_s_* and *w_e_* are the initial and ending values of the inertia weights, respectively, and *t* and *T* are the current and maximum iteration times.

The objective function of the identification is the root mean square error of the difference between the BW model displacement and the actual displacement, which is given as

(15) $$J(k,\alpha ,\beta ,\gamma )=\sqrt{\sum_{i=1}^{n}{\left(x_{i}-x_{i}^{h}\right)}^{2}/n}$$

where *x_i_* and *x_i_^h^* denote the actual measured displacement and model output displacement, respectively, and *n* denotes the number of samples. Based on the HGAPSO algorithm, the optimal parameters of the BW model can be derived.

## 4. Platform Open-Loop Test and Tracking Experiment

### 4.1. Test and Control System Construction

**Figure 6:**
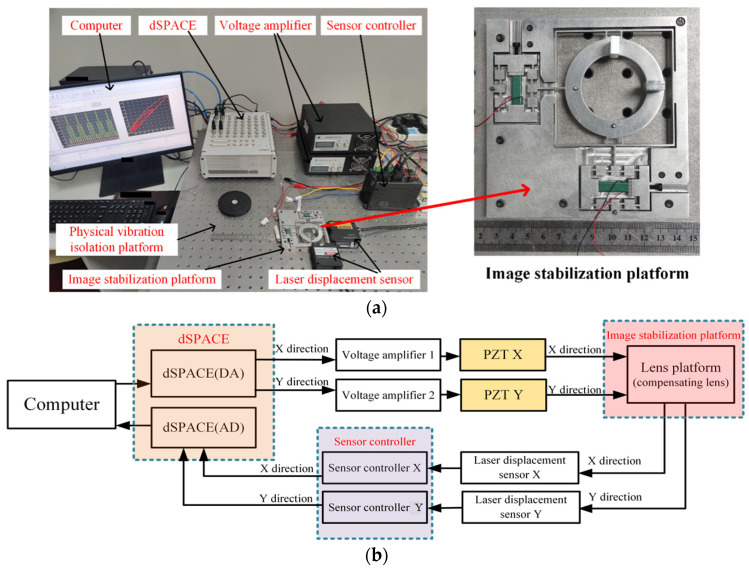
Test system. (**a**) Photograph of the test platform; (**b**) working principle flow diagram.

As shown in Figure 6a, the test and control system of the optical stabilization platform was constructed. And the whole experiment was carried out on a physically isolated vibration isolation table to avoid external interference. The system consists of a computer, an optical stabilization platform prototype, a dSPACE system, two single-channel voltage amplifiers, laser displacement sensors (LK-H025, LK-H008W, Keyence Philippines Inc., Osaka, Japan) and their controllers (LK-G5001, Keyence Philippines Inc., Japan). The sampling period of both laser displacement sensors is set to 50 kHz, and the measurement range meets the required image shift compensation range, while the linearity and repeatability of the laser displacement sensors can meet the submicron image shift compensation accuracy.

As depicted in Figure 6b, the system operates through the following closed-loop workflow. Digital signals of specific waveforms are generated by A user-written program in MATLAB 2013a software and converted to analog signals via dSPACE. These analog signals are then amplified by two single-channel piezoelectric amplifiers and applied to the corresponding PZTs within the piezoelectric optical stabilization platform. This excitation drives the central mechanism to produce linear displacement. Concurrently, a laser displacement sensor detects motion in the X and Y directions, converts the measured displacements into proportional electrical signals, and transmits them through its controller back to dSPACE for real-time processing, thereby closing the control loop.

### 4.2. Open Loop Testing and Performance Evaluation

(1) Stroke and coupling characteristics test

**Figure 7:**
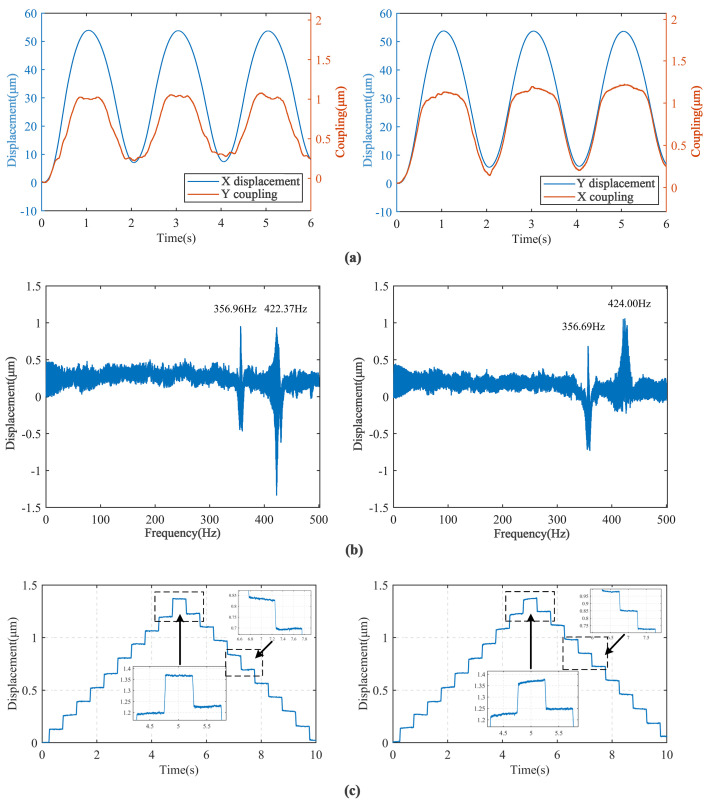
Open-loop test results. (**a**) Maximum travel and coupling displacement test; (**b**) intrinsic frequency test; (**c**) resolution test. X-axis result and Y-axis result sequentially.

The maximum travel and coupling displacement tests are conducted by applying 150 V to X or Y axis while maintaining 0 V on the orthogonal axis (Figure 7a). The platform demonstrates maximum travel ranges of 53.76 μm (X-axis) and 53.92 μm (Y-axis), with corresponding cross-axis coupling displacements limited to 1.23 μm (2.28%) and 1.07 μm (1.98%), respectively. These coupling ratios, well below the 3% operational threshold, confirm effective motion decoupling through the orthogonal guidance mechanism.

(2) Intrinsic frequency test

Frequency response analysis is performed using 2 V sinusoidal sweep signals across 0.1–500 Hz (Figure 7b). The system exhibits the first three resonant modes at 356.96 Hz (fundamental), 422.37 Hz (X-axis), and 424.00 Hz (Y-axis). The close proximity of orthogonal translational modes (Δ*f* = 1.63 Hz) and high fundamental frequency (>350 Hz) indicate well-balanced dynamic characteristics, enabling effective image stabilization across a broad operational bandwidth.

(3) Resolution test

To quantify the positioning resolution, stepwise triangular voltage signals (0–3 V) are applied to the platform (see Figure 7c). The results show that displacement values corresponding to the same voltage level differ between the rising and falling phases due to hysteresis. Displacement fluctuations are also observed in some step segments, attributable to noise from the voltage amplifier and position sensor. The maximum fluctuation means to a minimum resolution of 30 nm for the platform, which meets submicron stabilization requirements.

### 4.3. Tracking Performance and Control Optimization

**Figure 8:**
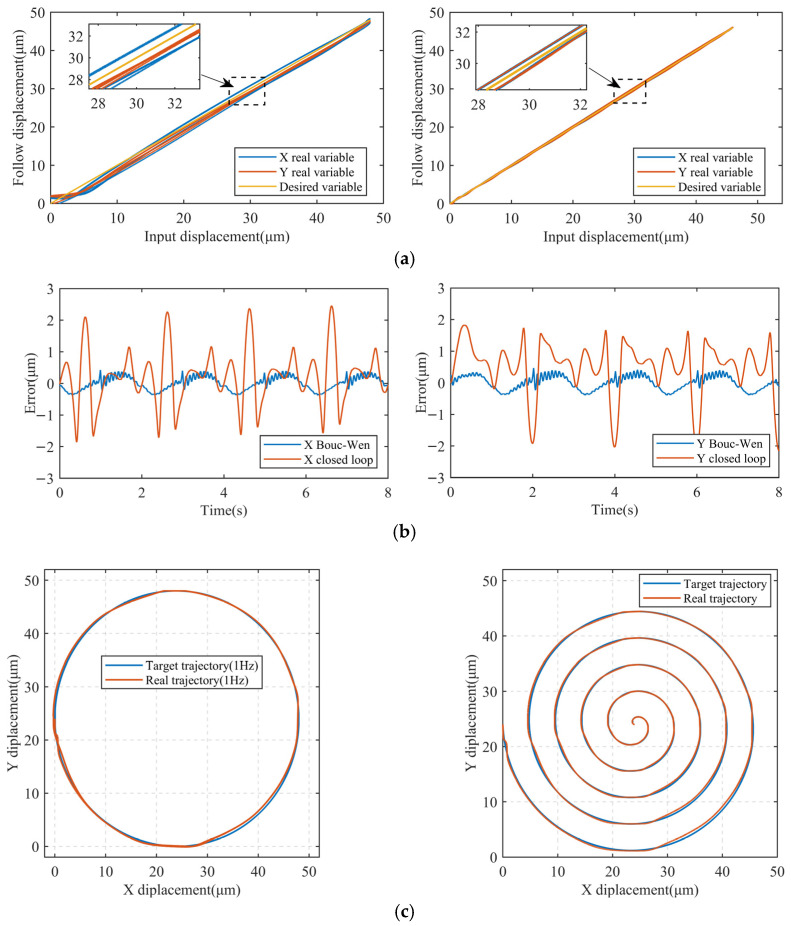
Tracking experimental results. (**a**) Feed-forward and composite control tracking; (**b**) error comparison of composite control strategies; (**c**) circular and spiral trajectory tracking.

The feed-forward controller is designed based on the optimal parameters identified by HGAPSO and tested by tracking a sinusoidal displacement signal with a maximum displacement of 48 μm (~130 V, 0.5 Hz) in both directions, as shown in Figure 8.

The results are shown in the left panel of Figure 8a, with maximum tracking errors of 2.23 μm (X) and 2.15 μm (Y) under the BW feed-forward control, representing 4.65% and 4.45% of the maximum displacement. Compared with the open-loop result, the hysteresis displacements in X/Y directions are reduced significantly. To further improve the tracking performance of the stabilized image platform, closed-loop tracking of the displacements is performed. The tracking results are shown in the right panel of Figure 8a.

The superior performance of the composite control strategy evident in Figure 8 can be attributed to its dual compensation mechanism. The HGAPSO-optimized BW feedforward component effectively cancels out the dominant, repeatable hysteresis nonlinearity of the PZT, which is the primary source of open-loop error. However, residual errors from unmodeled dynamics, minor creep effects, sensor noise, and external disturbances persist. The closed-loop PID feedback component actively suppresses these remaining uncertainties and disturbances, thereby achieving a further reduction in tracking error by approximately 80% (from ~2.2 µm to ~0.45 µm). The error curves in Figure 8b show that the composite control maintains a consistently low error band, indicating robust stability and disturbance rejection.

The successful tracking of both circular and spiral trajectories (Figure 8c) at 1 Hz demonstrates the platform’s effective motion decoupling and dynamic coordination in the XY plane. The slight deviation between the desired and actual paths is primarily due to the phase lag introduced by the PID controller at this frequency, which is a known trade-off for stability. Nevertheless, the tracking accuracy remains within the submicron range, confirming the platform’s capability to compensate for complex, time-varying image shift patterns encountered in real dynamic environments.

**Figure 9:**
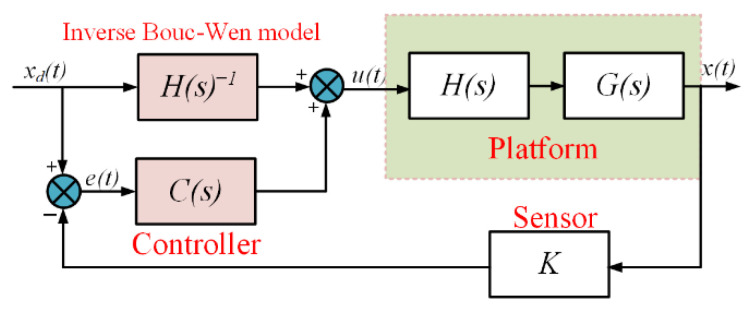
Composite control structure diagram.

In order to further improve the tracking performance of the device, a composite control method (Figure 9) is designed. The method introduces a closed-loop controller C(s) and a displacement sensing link K on the basis of feed-forward control. A PID controller was selected for C(s) due to its simplicity, reliability, and proven effectiveness in precision motion control. Its parameters were tuned on the experimental setup using a step-response method to achieve a balance between rapid response and stability, establishing a robust feedback baseline. This PID controller is used to realize the closed-loop control of the C(s) link to achieve more accurate control. From the tracking results of the composite closed-loop control (Figure 8b), the maximum tracking errors in both directions are 0.43 μm (X) and 0.47 μm (Y), which account for 0.90% and 0.98% of the maximum tracking displacement. By comparing the tracking errors of the feed-forward control and the composite control, it can be seen that the composite control is able to reduce the tracking error of the feed-forward compensation of the BW model to a larger extent and achieve the submicron tracking error of the platform.

Finally, the X and Y directions of the combined platform are used to track both circular and spiral image shift trajectories at 1 Hz to better verify the overall tracking performance of the platform, as shown in Figure 8c. The results show that the OIS platform decouples the motion in both X and Y directions through composite PID control, which can cope with the change in the image shift direction and accurately track the image shift motion of the planar trajectory.

## 5. Discussion

Experimental measurements reveal a 0.61% (X-axis) and 1.03% (Y-axis) reduction in maximum travel compared to theoretical predictions, with values marginally lower than finite element analysis (FEA) results. This deviation originates from four primary factors: (a) the presence of chamfers within 0.2 mm in the flexible hinge and guide mechanism of the prototype, and a non-ideal right-angle hinge, which resulted in a large input stiffness, thus reducing the total travel; (b) the actual measurements, in which the contact area between the PZT and the mechanism was smaller than the assumptions made in the theory and the FEA, and the use of the pre-tensioning of the screws and the shims, which introduced an additional error; (c) the mechanism machining of the non-perfect symmetry further affected the results; and (d) the measurement was carried out with an aluminum block as the measurement point of the sensor, whose additional mass had an impact on the maximum travel. Despite these discrepancies, the close agreement between experimental and simulated results validates the design’s theoretical foundation.

Based on Figure 3d and Figure 7b, the first three orders of the platform’s intrinsic frequency are reduced by 7.63%, 7.92 and 8.14%, respectively, compared with the simulation results. These differences stem from the coupled effects: (a) the effects of PZT and spacer mass are not considered in the simulation, and the increase in the equivalent mass of the platform during the actual motion leads to the reduction in the resonant frequency. (b) The equivalent stiffness of the actual platform is larger than that assumed in the FEA, which further leads to the reduction in the resonant frequency. These factors work together to make the experimental results deviate from the simulation results.

In practical applications, mounting an optical component (e.g., a compensation lens with a typical mass of 5–20 g) will slightly increase the effective mass of the system. As shown in the load capacity simulation in Figure 3e, a 2 N vertical load induces only 0.236 µm of out-of-plane displacement, which corresponds to merely 0.6% of the full in-plane stroke. The static amplification ratio remains essentially unchanged due to the high in-plane stiffness of the guiding beams, and the expected shift in the first resonant frequency is estimated to be less than 2%. Given that the resulting force and dynamic in-fluence are minimal, the difference between unloaded and loaded operation is negligible for the scope of this study.

Based on Figure 8, the composite PID based on the HGAPSO identified BW feed-forward model can effectively compensate for the hysteresis displacement caused by PZT. The results of trajectory tracking show that the optically stabilized image platform exhibits good displacement repeatability under low-frequency conditions and achieves submicron positioning and tracking accuracy. However, limited by the performance of the PID algorithm, there is still room for improvement of the platform’s control ability under high-frequency trajectories. Furthermore, residual tracking errors observed during complex planar motions indicate the influence of dynamic cross-axis coupling. While the current composite controller mitigates static nonlinearities, advanced decoupling control strategies (e.g., model-based multivariable control or dynamic feed-forward compensation) will be investigated in future work to further enhance trajectory tracking performance. In the next step, the control algorithm will be optimized to further improve the positioning and tracking performance under high-frequency conditions, so as to enhance the platform’s dynamic response capability and image stabilization effect.

## 6. Conclusions

This study presents a piezoelectric-driven XY optical stabilization platform designed to compensate for linear image shifts in planar coordinates through integrated mechanical design and advanced control strategies.

(1) A comprehensive kinematic evaluation of the platform was conducted by establishing an accurate mathematical model and performing simulations and experimental validations.
(2) To improve the positioning and tracking accuracy of the platform, the hysteresis model of PZT is identified by using the HGAPSO algorithm, and the strategy of BW feed-forward model combined with composite PID control is designed. The results show that the platform achieves a motion range of 53.92 μm × 53.76 μm with a maximum resolution of 30 nm and submicron tracking control under low-frequency conditions.
(3) The achievements provide an important reference and guidance for the design and high-frequency control of steady image platforms.

This work lays a foundation for extending the platform’s operational bandwidth through adaptive control algorithms in complex vibration environments. While the current system demonstrates high-precision tracking under well-controlled conditions, further tests with broadband random vibrations and multi-axis coupled perturbations will be conducted in the future. We will focus on enhancing its performance in real-world dynamic environments, improving the linearity of the structure, and extending the working stroke of the displacement amplifier. Additionally, the nonlinear effects of the central platform will be systematically analyzed through high-precision modeling and compensated for via hybrid control strategies to achieve a more linear input-output relationship. These improvements will address the current limitations and provide a foundation for next-generation high-precision stabilization systems.

## Figures and Tables

**Table 1:** Platform design indicators.

Maximum Size (mm)	Travel Range (μm)	Intrinsic Frequency (Hz)	Parasitic Coupling	Positioning Accuracy (μm)
130 × 130 × 20	≥50 × 50	≥300	≤2.5%	<1

**Table 2:** Material properties.

Material	Yield Strength (Mpa)	Young’s Modulus (Gpa)	Density (g/cm^3^)	Poisson’s Ratio
7075Al	503	71	2.81	0.33

**Table 3:** PZT performance parameters.

Model	Dimension (mm)	Drive Voltage (V)	Maximum Displacement (μm)	Stiffness (N/μm)	Resonant Frequency (KHz)	Thrust (N)
Pst150/5 × 5/20H	5.1 × 5.1 × 18	0~150	20	60	50	1600

**Table 4:** Platform structural parameters.

Parameter	*t*	*b*_0_	*b*_1_	*b*_2_	*t*_d_	*l*_1_,*l*_2_
Value (mm)	1	13	2.05	6	0.9	6
Parameter	*l*_3_	*l*	*l_a_*	*l_b_*	*l_c_*	*l*_d_
Value (mm)	2.5	3	27	30	3.3	17

## Data Availability

The data that support the findings of this study are available from the corresponding author upon reasonable request.
